# Genetic Diversity and Population Structure in Two Mangrove Species (*Sonneratia alba* and *Sonneratia caseolaris*) Across Coastal Areas of Thailand

**DOI:** 10.3390/biology15020141

**Published:** 2026-01-13

**Authors:** Supaporn Khanbo, Chaiwat Naktang, Wasitthee Kongkachana, Chutintorn Yundaeng, Nukoon Jomchai, Nattapol Narong, Tamanai Pravinvongvuthi, Pasin Maprasop, Waratthaya Promchoo, Sithichoke Tangphatsornruang, Wirulda Pootakham

**Affiliations:** 1 National Omics Center, National Center for Genetic Engineering and Biotechnology, National Science and Technology Development Agency (NSTDA), Pathum Thani 12120, Thailand; 2 Department of Marine and Coastal Resources, 120 The Government Complex, Chaengwatthana Rd., Thung Song Hong, Bangkok 10210, Thailand

**Keywords:** *Sonneratia alba*, *Sonneratia caseolaris*, single-nucleotide polymorphism, population structure, genetic diversity, mangrove forest, RAD-seq

## Abstract

*Sonneratia alba* Sm. and *Sonneratia caseolaris* (L.) Engl. are two ecologically important components of mangrove communities in Thailand. However, their population genetic patterns in Thailand remain poorly understood. Here, we assessed the genetic diversity and population structure of 107 *S. alba* and 131 *S. caseolaris* individuals sampled across their full coastal range in Thailand using single-nucleotide polymorphism (SNP) markers. Population structure analyses consistently revealed strong genetic subdivision associated with geography: *S. alba* formed three clusters (including one admixed group), whereas *S. caseolaris* formed two clusters. In both species, populations were clearly separated between the Andaman Sea and the Gulf of Thailand, reflecting the isolating influence of the Malay Peninsula. Genetic differentiation between clusters was high (*F*_ST_ = 0.364 in *S. alba* and 0.321 in *S. caseolaris*). Genetic differentiation increased with geographic distance in *S. caseolaris*, whereas no such relationship was detected in *S. alba*. Both species exhibited low levels of genetic diversity (H_o_ = 0.173; H_e_ = 0.223 in *S. alba* and H_o_ = 0.290; H_e_ = 0.406 in *S. caseolaris*). Together, these results reveal pronounced spatial genetic structure and limited evolutionary connectivity between coastal regions, providing genome-wide insights into mangrove population differentiation with important implications for conservation and restoration.

## Simple Summary

To assess the genetic diversity and population structure of *Sonneratia alba* and *Sonneratia caseolaris* in Thailand, we used restriction site-associated DNA sequencing (RAD-seq) to genotype individuals from natural populations along the Thai coast. Based on SNPs identified from the *S. alba* and *S. caseolaris* genome sequences, we found low genetic diversity and high genetic differentiation among 107 *S. alba* and 131 *S. caseolaris* individuals. Population structure was strongly associated with geography: individuals formed two main genetic clusters corresponding to the Andaman Sea and Gulf of Thailand coasts, as consistently supported by population structure, principal component and phylogenetic analyses.

## 1. Introduction

Mangrove forests are a key component of coastal ecosystems across tropical and subtropical regions worldwide [1]. They cover approximately 14.7 million hectares and comprise 83 true mangrove species (including 12 hybrids) along with more than 100 associated species [2]. Mangrove forests are among the most important and highly productive coastal ecosystems [3]. They provide numerous ecological functions, including storm surge protection and carbon sequestration for climate change mitigation [4,5]. Mangroves also serve as critical habitats for a wide range of fauna and offer multiple ecosystem services that support coastal communities [6]. In addition, they provide economically valuable resources, such as timber and medicines [7]. Despite their importance, mangroves are vulnerable to various anthropogenic disturbances, including overexploitation, land reclamation, freshwater diversion for irrigation, infrastructure development, and urbanization [8,9]. These activities contribute to habitat loss and erode genetic diversity within mangrove species by reducing effective population sizes, which promotes inbreeding and accelerates diversity loss through genetic drift [10,11,12]. As genetic variation declines, populations may suffer reduced fitness and adaptive potential, increasing extinction risk.

In Thailand, mangrove forests are primarily distributed along the coastlines of the Andaman Sea and the Gulf of Thailand. Mangrove forests are also found on muddy tidal flats at the river mouths [13,14]. Coastal differences in monsoon regimes, currents, and estuarine conditions shape propagule dispersal and the population genetic structure of mangrove populations. According to recent global data on mangrove extent [15], approximately 23% of Thailand’s mangrove forests were lost between 1970 and 2020 along both coastlines [16,17], with the most significant mangrove area losses occurring before 2000. These declines require the implementation of effective strategies for the protection, conservation, and restoration of mangrove ecosystems [18].

The mangrove genus *Sonneratia* is one of the dominant and ecologically important genera in Thailand [19]. It comprises six species and is widely distributed throughout the Indo-West Pacific region [20]. *Sonneratia caseolaris* (L.) Engl. is a widespread species, ranging from Sri Lanka through the Malay Peninsula to China and Australia [21,22]. It can naturally grow in both saltwater and freshwater environments [23]. Its closely related species, *Sonneratia alba* Sm., is distributed from East Africa through Southeast Asia to southern Japan and northeastern Australia [24]. *S. alba* is highly tolerant to salinity and typically dominates zones along the seaward fringe of mangrove ecosystems [25,26]. In Thailand, *S. caseolaris* is locally known as Lampoo, while *S. alba* is referred to as Lampoo Thale [19]. Both species are key structural components of estuarine mangrove forests and provide important ecological and coastal protection services. Although *S. alba* and *S. caseolaris* are not currently listed as threatened [12], continued deforestation and degradation of mangrove habitats may lead to long-term genetic risks. Previous studies on the genetic diversity and population structure of *S. alba* using chloroplast fragments and nuclear genes have shown that this species exhibits low within-population genetic diversity and pronounced genetic structure across the Indo–West Pacific [24]. Wee et al. [27] further characterized the genetic structure and gene flow of *S. alba* using nuclear microsatellite markers and reported significant east–west genetic differentiation across the Malay Peninsula. In contrast, *S. caseolaris* populations in Vietnam, evaluated using ISSR markers, exhibit moderate-to-high levels of genetic diversity and cluster into three genetic groups corresponding to their geographic distribution [28]. However, existing knowledge of both species is mainly based on organelle markers or a limited number of nuclear loci. These marker systems provide insufficient resolution for detecting contemporary gene flow, cryptic subdivision, and subtle population divergence that are critical for conservation planning.

In addition to population genetic structure, several studies in mangrove species have reported genetic variation associated with adaptive traits such as salinity tolerance, growth performance, and responses to environmental stress, primarily using candidate gene approaches or targeted molecular markers [26,29]. While these studies have provided important insights into local adaptation, they were generally limited to a small number of loci and did not assess how such trait-associated variation is distributed across natural populations or structured by geographic barriers. Consequently, the extent to which adaptive potential is shaped by coastal configuration and evolutionary connectivity remains poorly understood in many mangrove taxa, including species of the genus *Sonneratia*.

A genome-wide approach offers a more powerful means of assessing coastal connectivity and identifying distinct evolutionary lineages. This approach enables genetic diversity and population structure to be examined within an explicit spatial and evolutionary framework that cannot be achieved using marker-limited methods. Among molecular markers, single-nucleotide polymorphisms (SNPs) have emerged as the most prevalent marker type in the postgenomic era due to their abundance, stability, and wide genomic distribution [30,31,32,33]. Although individual SNPs contain less information than microsatellites, they are routinely applied in large panels containing thousands of loci, providing greater precision for estimating genetic diversity and enabling more robust assessments of local adaptation [34,35]. 

High-resolution SNP datasets generated through restriction site-associated DNA (RAD) sequencing allow quantification of genome-wide variation, reveal fine-scale genetic structure, and support evidence-based restoration strategies [36,37]. SNP markers have already been successfully used to evaluate the population genetics of several mangrove species [30,38,39,40,41]. For *S. alba* and *S. caseolaris*, however, population structure and genetic diversity have never been examined using genome-wide SNP markers. Such information is essential because restoration programs in Thailand frequently overlook genetic provenance, risking the loss of locally adapted genotypes and potentially reducing long-term ecosystem resilience. Incorporating genomic evidence into mangrove restoration is therefore critical for maintaining adaptive potential under rapid coastal change.

In this study, we evaluated the genetic diversity and population structure of two mangrove species, *S. alba* and *S. caseolaris*, along the coastlines of Thailand using high-quality SNP markers generated through RAD sequencing. By applying a genome-wide SNP approach, this study provides the first comparative population genomic assessment of these two species in this region. Our findings elucidate patterns of evolutionary connectivity and spatial genetic structure, with implications for mangrove conservation and restoration efforts.

## 2. Materials and Methods

### 2.1. Sample Collection

**Figure 1:**
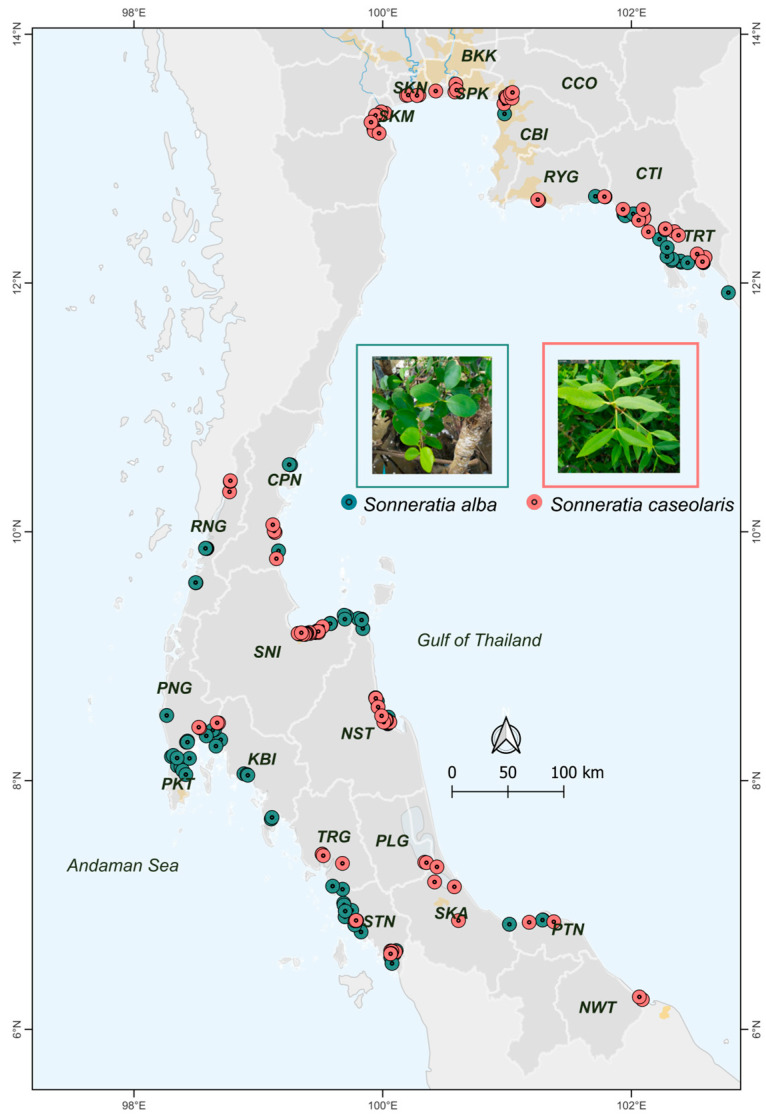
Sampling locations of *S. alba* and *S. caseolaris* in Thailand. *S. alba* was sampled from 15 provinces and *S. caseolaris* from 21 provinces along the Andaman Sea and Gulf of Thailand coasts. Province abbreviations are defined in Supplementary Tables S1 and S2. Dots denote sampled individuals at each site.

A total of 107 *S. alba* and 131 *S. caseolaris* individuals were sampled from mangrove forests along the Andaman Sea and the Gulf of Thailand coasts (Figure 1; Tables S1 and S2). For *S. alba*, leaf tissue was collected from 15 provinces in Thailand (Figure 1; Table S1): Chon Buri (CBI; n = 1), Chumphon (CPN; n = 5), Chanthaburi (CTI; n = 10), Krabi (KBI; n = 7), Nakhon Si Thammarat (NST; n = 12), Phuket (PKT; n = 5), Phang-nga (PNG; n = 10), Pattani (PTN; n = 2), Ranong (RNG; n = 6), Rayong (RYG; n = 3), Songkhla (SKA; n = 4), Surat Thani (SNI; n = 14), Satun (STN; n = 12), Trang (TRG; n = 3), and Trat (TRT; n = 13). For *S. caseolaris*, leaf tissue was collected from 21 provinces (Figure 1; Table S2): Bangkok (BKK; n = 1), Chon Buri (CBI; n = 1), Chachoengsao (CCO; n = 6), Chumphon (CPN; n = 5), Chanthaburi (CTI; n = 10), Nakhon Si Thammarat (NST; n = 14), Narathiwat (NWT; n = 2), Phetchaburi (PBI; n = 5), Phatthalung (PLG; n = 2), Phang-nga (PNG; n = 5), Pattani (PTN; n = 2), Ranong (RNG; n = 10), Rayong (RYG; n = 3), Songkhla (SKA; n = 3), Samut Songkhram (SKM; n = 5), Samut Sakhon (SKN; n = 5), Samut Prakan (SPK; n = 3), Surat Thani (SNI; n = 15), Satun (STN; n = 21), Trang (TRG; n = 5), and Trat (TRT; n = 8). Sites on the Gulf of Thailand include BKK, CBI, CCO, CPN, CTI, NST, NWT, PBI, PLG, RYG, SKA, SKM, SKN, SPK, SNI, PTN, and TRT, whereas Andaman Sea sites include KBI, PKT, PNG, RNG, STN, and TRG. Only young, healthy leaf samples were collected from mature trees, with a minimum spacing of at least 20 m between individuals to minimize resampling of ramets or close relatives. Sample sizes varied among sites according to local population sizes. All samples were collected during 2024–2025. Sampling locations were mapped in QGIS v3.24.2 (Figure 1).

### 2.2. DNA Extraction, RAD-seq Library Preparation and Sequencing

Genomic DNA was extracted from young leaves using the CTAB method [42]. DNA was further purified with the DNeasy Plant Mini Kit (Qiagen, Hilden, Germany) according to the manufacturer’s instructions and quantified on a Qubit fluorometer (Thermo Fisher Scientific, Waltham, MA, USA) using the dsDNA BR Assay kit (Thermo Fisher Scientific, Waltham, MA, USA).

RAD-seq libraries were prepared from 1 µg of input DNA per sample using the MGIEasy RAD Library Prep Kit (MGI Tech, Shenzhen, China) following the manufacturer’s instructions. Briefly, genomic DNA was digested with TaqI and fragments were ligated to unique barcoded adapters. The samples were pooled in equimolar amounts and subjected to PCR amplification. Libraries were quantified and quality-checked on a Fragment Analyzer system (Agilent Technologies, Santa Clara, CA, USA) using an NGS Fragment Analysis kit. Sequencing was performed on an MGISEQ-2000RS platform (paired-end 150 bp; MGI Tech, Shenzhen, China) following the manufacturer’s protocol.

### 2.3. Single-Nucleotide Polymorphisms Identification

Raw sequence data were processed with the Genome Analysis Toolkit (GATK) v4.1.4.1 pipeline [43]. Paired-end, quality-filtered reads from *S. alba* were aligned to the *S. alba* reference genome (DDBJ/EMBL/GenBank accession PRJEB8424), and reads from *S. caseolaris* were aligned to the *S. caseolaris* reference (Genome Warehouse (GWH) of NGDC accession GWHBCIR00000000) using BWA v0.7.19 -r1273 (https://github.com/lh3/bwa, accessed on 12 July 2025) with default parameters. Sequence alignment map (SAM) files were converted to binary format (BAM), then sorted and indexed using samtools v1.9 [44]. Mapping statistics were also obtained with samtools. SNPs were called using GATK v4.1.4.1 with HaplotypeCaller [43] and filtered using the following thresholds: (i) minor allele frequency (MAF) > 0.05; (ii) depth of coverage between 10× and 200×; and (iii) no missing genotypes. The filtered SNP sets were used for downstream analyses of genetic diversity and population structure in each species.

### 2.4. Population Genetic Structure Analysis

Genetic clustering for each species was evaluated using a Bayesian model-based approach implemented in STRUCTURE v2.3.4 [45]. We tested K = 1–10 with 20 replicate runs per K under the admixture model with the correlated allele frequencies; each run used a burn-in of 100,000 followed by 1,000,000 MCMC iterations. The optimal K was determined from ΔK [46] and the mean log posterior probability [lnP(D)], as implemented in STRUCTURE Harvester v0.6.94 [47]. Based on the optimal K value, clustering from 10,000 replicates in STRUCTURE was summarized with CLUMPP v1.1.2 [48] to obtain average cluster membership proportions. In addition, principal component analysis (PCA) was used to visualize genetic structure within each species. PCA was performed using the PCA function in R. The first two principal components were selected and plotted using R software v3.3.4 with the library ggplot2 v3.5.0 [49]. To further explore population clustering within each species, unrooted consensus neighbour-joining (NJ) trees were inferred using MEGA11 [50]. A bootstrap consensus tree with 1000 replications was performed. The NJ trees were visualized with iTOL v6 [51].

Analysis of molecular variance (AMOVA) among populations within each species was performed in Arlequin v.3.5 [52] with 10,000 permutations to estimate genetic variance within and among populations based on the results of the clustering analyses. Population differentiation (pairwise *F*_ST_) among STRUCTURE-inferred clusters and among sampling sites was also estimated with Arlequin v3.5 [52] and visualized in R v3.3.4 using the ggplot2 package v3.5.0 [49]. To reduce bias from small sample sizes, locations represented by a single individual were excluded from the *F*_ST_ analysis.

### 2.5. Isolation-by-Distance Analysis (Mantel Test)

To determine whether geographic distance explained patterns of genetic differentiation (isolation by distance, IBD) along each coastline in *S. alba* and *S. caseolaris*, we conducted a Mantel test in GenAlEx v6.5 [53] with 9999 permutations. Mantel tests were performed separately for the Andaman Sea and Gulf of Thailand populations. Pairwise geographic distances among sampling sites were compared with pairwise genetic distances [*F*_ST_/(1 − *F*_ST_)] calculated at the site (population) level.

### 2.6. Genetic Diversity Analysis

To estimate genetic diversity at the population and species levels, we computed the number of effective alleles (N_e_), Shannon’s information index (I), observed heterozygosity (H_o_), expected heterozygosity (H_e_) and the percentage of polymorphic loci (PPL) for each group using GenAlEx v6.502 [53]. The inbreeding coefficient (*F*_IS_) for each population was also calculated in GenAlEx v6.502. Gene flow (N_m_) among groups was approximated as Nm = [(1/*F*_ST_) − 1]/4 [54]. Polymorphism information content (PIC) values for SNP markers were calculated using PowerMarker v3.25 [55].

## 3. Results

### 3.1. Sequencing and SNP Discovery

A total of 107 individuals of *S. alba* and 131 of *S. caseolaris* were genotyped using RAD-seq. Sequencing produced 2,001,629,636 and 2,974,679,250 reads for *S. alba* and *S. caseolaris*, respectively, of which 89.78% and 92.83% mapped to their respective reference genomes. Average read counts per individual were 18,706,819 for *S. alba* (Table S1) and 22,707,475 for *S. caseolaris* (Table S2). Initial SNP calling identified 5,059,036 and 4,696,679 putative loci in *S. alba* and *S. caseolaris*, respectively. After applying filtering thresholds, a total of 2664 SNPs for *S. alba* (Table S3) and 5208 SNPs for *S. caseolaris* (Table S4) were retained for downstream analyses. Polymorphic information content (PIC) was estimated for each SNP to quantify marker informativeness across 107 *S. alba* and 131 *S. caseolaris* individuals. In *S. alba*, PIC values ranged from 0.09 to 0.58 (mean = 0.19), whereas in *S. caseolaris*, PIC values ranged from 0.27 to 0.38 (mean = 0.34) (Figure S1).

To further characterize genome-wide patterns of genetic variation, we examined the chromosomal distribution and functional positions of RAD-seq SNPs in both species. In *S. alba*, SNPs were identified across all chromosomes, with the number of variants per chromosome ranging from 144 to 335 (Table S5). Similarly, in *S. caseolaris*, SNPs were detected on all chromosomes, with variant counts ranging from 281 to 636 per chromosome (Table S6). To explore patterns of SNP distribution in both species, SNP frequency was plotted at 1 Mb intervals along each chromosome. SNP density across the genome ranged from 0 to 50 SNPs per megabase (Mb), with an average density of approximately one SNP per 75 kb in *S. alba* and one SNP per 36 kb in *S. caseolaris*. Although the number of SNPs varied among chromosomes and between species, both datasets exhibited broad genome-wide coverage rather than concentration on a limited number of chromosomes (Figure S2). Regions of chromosomes lacking SNPs generally corresponded to areas where no RAD-seq reads were recovered, rather than indicating an absence of polymorphism.

Functional annotation of SNPs revealed similar genomic distributions in both species. In *S. alba*, most SNPs were located in downstream (34.41%) and upstream (30.49%) regions, followed by exonic (13.99%) and intronic (10.76%) regions, with smaller proportions occurring in intergenic regions and splice-related sites. A comparable pattern was observed in *S. caseolaris*, where downstream (32.41%) and upstream (32.15%) regions contained the largest fraction of SNPs, followed by intronic (10.35%) and exonic (11.43%) regions. Because SNP calling was performed independently for each species using species-specific reference genomes, direct positional comparisons of SNPs between *S. alba* and *S. caseolaris* were not conducted. Accordingly, comparisons focused on overall genome-wide patterns of SNP distribution and functional category composition rather than one-to-one positional correspondence between species.

The RAD-seq approach applied in this study was not designed to specifically target previously reported candidate genes associated with adaptive traits. Consequently, direct detection of such trait-associated variants could not be confirmed, and coverage of these genomic regions cannot be inferred from the present dataset.

### 3.2. Population Genetic Structure

**Figure 2:**
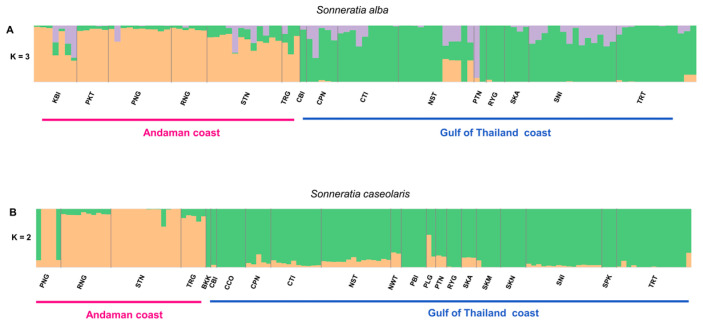
Genetic structure of (**A**) *S. alba* and (**B**) *S. caseolaris* in Thailand inferred with STRUCTURE. Each vertical bar represents one individual; colour proportions within bars denote ancestry membership coefficients (Q). For *S. alba*, representative results for K = 3 are shown in orange (cluster 1), green (cluster 2), and purple (cluster 3); for *S. caseolaris*, K = 2 is shown in orange (cluster 1) and green (cluster 2) (the ΔK-supported value). Individuals are ordered by province along the Andaman Sea and Gulf of Thailand coasts; vertical black lines indicate province boundaries.

**Figure 3:**
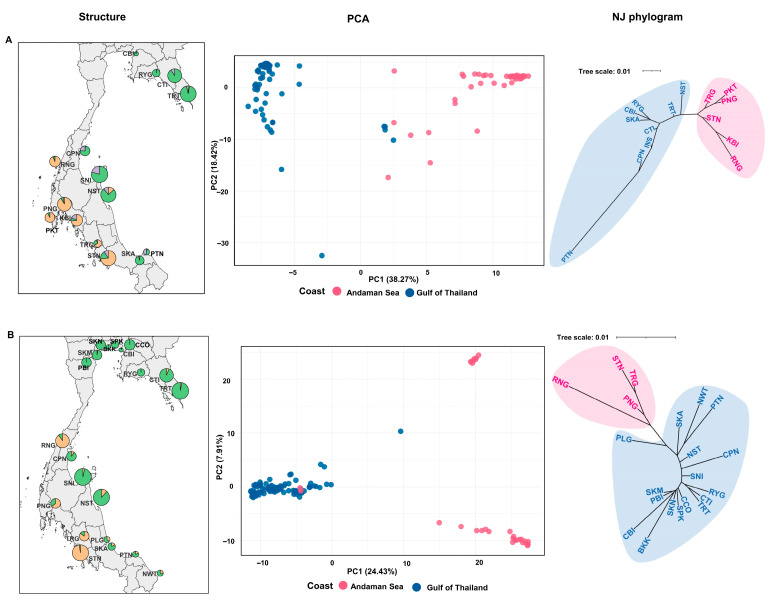
Genetic clustering across Thailand’s coasts for (**A**) *S. alba* and (**B**) *S. caseolaris*. (**Left**) STRUCTURE summaries mapped by population; each circle marks a sampling site, circle size is proportional to the number of individuals, and segment colours denote the mean ancestry coefficients (Q) per site at the ΔK-optimal K value (orange = cluster 1; green = cluster 2). (**Center**) PCA of individuals based on SNP genotypes; points are colour-coded by coast (Andaman Sea vs. Gulf of Thailand) and axis labels indicate the percentage of variance explained. (**Right**) Unrooted neighbour-joining (NJ) networks based on Nei’s genetic distance among populations; Shaded regions denote population groupings by coast (blue = Gulf of Thailand; pink = Andaman Sea). Province abbreviations follow Supplementary Tables S1 and S2.

To understand the population structure of *S. alba* and *S. caseolaris* in Thailand, three different approaches—STRUCTURE, PCA and a neighbour-joining (NJ) tree—were applied. All analyses used the SNP genotype sets of 2664 loci for *S. alba* and 5208 for *S. caseolaris*. In STRUCTURE, the number of clusters was determined using the ΔK method [46]. The ΔK distribution showed clear modes at K = 3 for *S. alba* and at K = 2 for *S. caseolaris* (Figure S3). Under the Evanno criterion, the STRUCTURE results provided the strongest support for three genetic clusters in *S. alba* and two clusters in *S. caseolaris* (Figure 2). In *S. alba*, at K = 3, ancestry coefficients revealed a clear coastal partition, with one cluster predominating along the Andaman Sea coast and another along the Gulf of Thailand coast. A third cluster appeared primarily as admixture across several sites. Populations from KBI, PKT, PNG, RNG, and STN (Andaman Sea) together with TRT and NST (Gulf of Thailand) formed one genetic cluster and shared inferred ancestry with individuals from both coasts (Figure 2A). For *S. caseolaris*, most individuals from the same geographic region were assigned to the same genetic cluster (Figure 2B). Cluster 1 (orange) was dominated by Andaman Sea samples (PNG, RNG, STN, TRG), whereas Cluster 2 (green) comprised mainly Gulf of Thailand samples (BKK, CCO, CPN, CTI, NST, NWT, PBI, PLG, PTN, RYG, SKA, SKM, SKN, SNI and TRT). Some individuals showed mixed ancestry, and a few, particularly those from PNG, grouped with the opposite coast, indicative of admixture or recent gene flow. Consistent with the STRUCTURE results, both the PCA and the NJ tree revealed a pronounced genetic divide between the Andaman and Gulf of Thailand coasts (Figure 3), with each cluster largely confined to one coast. The first two principal components explained 55.65% and 32.34% of the total genetic variance in *S. alba* and *S. caseolaris*, respectively. The NJ trees of both species also revealed two major clades corresponding to the two coastal regions, consistent with the STRUCTURE and PCA results (Figure 3).

### 3.3. Genetic Differentiation of Populations

We quantified genetic differentiation (*F*_ST_) among STRUCTURE-defined subpopulations to assess relationships among groups. Following standard thresholds [56], *F*_ST_ values of 0.00–0.05 indicate low differentiation, 0.05–0.15 indicate moderate differentiation, and > 0.15 indicate high differentiation. Genetic differentiation among STRUCTURE-defined populations was high in both species (*S. alba*: *F*_ST_ = 0.364, *p* < 0.001; *S. caseolaris*: *F*_ST_ = 0.321, *p* < 0.001). The mean number of migrants per generation (N_m_), estimated from *F*_ST_ as N_m_ = [(1/*F*_ST_) − 1]/4, was 0.437 for *S. alba* and 0.528 for *S. caseolaris*. Values of N_m_ < 1 indicate limited gene flow, consistent with the high *F*_ST_ values, and together these results point to substantial population divergence in both species. Pairwise *F*_ST_ estimates indicated high differentiation, ranging from 0.296 to 0.412 for *S. alba* and 0.321 for *S. caseolaris* (two-group comparison; Table S7). In *S. alba*, the greatest divergence occurred between subpopulation 1 and subpopulation 2 (*F*_ST_ = 0.412). AMOVA revealed significant genetic differentiation among STRUCTURE-defined populations in both species (*p* < 0.01). In *S. alba* (K = 3), 36.43% of the variance was partitioned among populations and 63.57% within populations. In *S. caseolaris* (K = 2), 32.15% of the variance occurred among populations and 67.85% within populations (Table 1). Overall, population structure is pronounced in both species, with *S. alba* exhibiting stronger differentiation than *S. caseolaris*.

**Figure 4:**
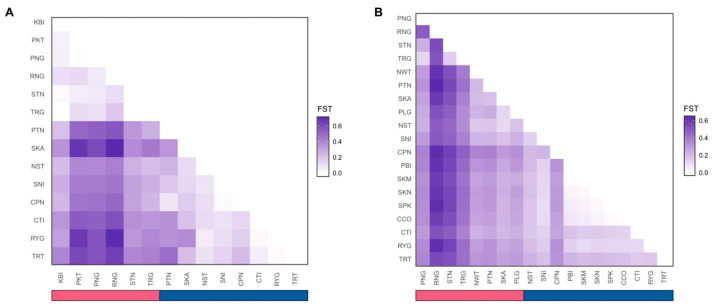
Pairwise genetic differentiation (*F*_ST_) heatmaps for (**A**) *S. alba* and (**B**) *S. caseolaris*. Site labels are ordered by province along the Andaman Sea (pink) and Gulf of Thailand (blue) coasts, as indicated by the colour bar. Sites with n = 1 were excluded from the analysis.

Population differentiation (pairwise *F*_ST_) among sampling locations was also estimated. For *S. alba*, pairwise *F*_ST_ ranged from 0.002 to 0.720 (Figure 4A and Table S8). The largest values were generally observed for between-coast comparisons of sampling sites, such as RNG and SKA, whereas the smallest values occurred within coasts, including RYG–TRT and TRG–KBI. For *S. caseolaris*, pairwise *F*_ST_ ranged from 0.007 to 0.651 (Figure 4B and Table S8). High differentiation was concentrated in between-coast comparisons of sampling sites, such as RNG–PTN, whereas within-coast pairs, such as PBI–SKM, showed the lowest values. Mean pairwise *F*_ST_ by coast pairing showed that, for *S. alba*, values were 0.075 within the Andaman Sea, 0.173 within the Gulf of Thailand, and 0.435 between coasts. For *S. caseolaris*, values were 0.348 within the Andaman Sea, 0.209 within the Gulf of Thailand, and 0.465 between coasts. In both species, the between-coast mean exceeded the within-coast means, consistent with strong coastal structure.

### 3.4. Correlation Analysis Between Genetic Distance and Geographic Distance

**Figure 5:**
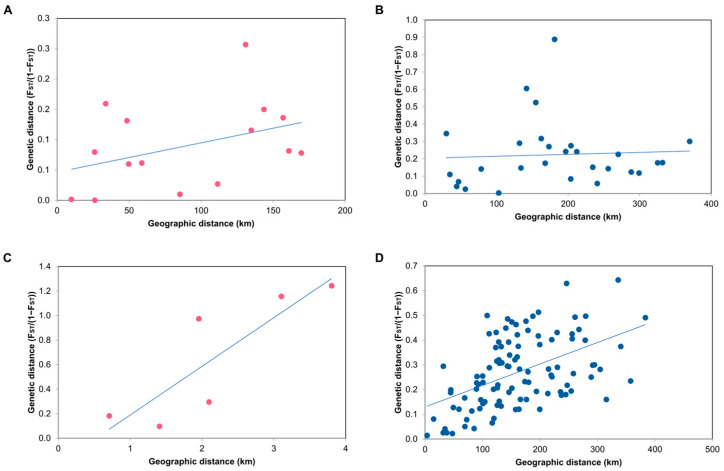
Scatterplot of Mantel test results showing the relationship between genetic differentiation and geographic distance for *S. alba* and *S. caseolaris*. Panels (**A**,**B**) show *S. alba* from the Andaman Sea coast ((**A**); R^2^ = 0.152, *p* = 0.066) and the Gulf of Thailand coast ((**B**); R^2^ = 0.003, *p* = 0.353). Panels (**C**,**D**) show *S. caseolaris* from the Andaman Sea coast ((**C**); R^2^ = 0.728, *p* = 0.044) and the Gulf of Thailand coast ((**D**); R^2^ = 0.247, *p* = 0.001). Points represent pairwise comparisons among sampling sites; the solid line indicates the fitted linear regression (trend) line. Points are coloured by coastline (pink = Andaman Sea; blue = Gulf of Thailand).

In order to explore the spatial genetic structure of populations, the Mantel test is commonly used to assess whether the species conform to the model of isolation by distance (IBD) by evaluating the correlation between genetic and geographic distances. Patterns of IBD differed between *S. alba* and *S. caseolaris* (Figure 5). For *S. alba*, Mantel tests showed no significant correlation between pairwise genetic distances and coastal geographic distances along either coastline. The Andaman Sea populations exhibited a weak, non-significant positive trend (R^2^ = 0.152; *p* = 0.066), while the Gulf of Thailand populations showed no relationship (R^2^ = 0.003; *p* = 0.353). These results indicate that geographic distance is not a major driver of genetic differentiation in *S. alba* across the sampled sites. In contrast, *S. caseolaris* exhibited clear evidence of IBD along both coasts. A strong and significant correlation was detected in the Andaman Sea populations (R^2^ = 0.728; *p* = 0.044), and a moderate but significant correlation was also observed among Gulf of Thailand populations (R^2^ = 0.247; *p* = 0.001). These results suggest that genetic divergence in *S. caseolaris* increases with coastal distance, implying more spatially restricted propagule dispersal and stronger geographic structuring relative to *S. alba*.

### 3.5. Genetic Diversity

Diversity statistics were computed for two coastal groupings (Andaman Sea vs. Gulf of Thailand) in each species (Table 2). For *S. alba*, mean values across the two coasts for the effective number of alleles (N_e_) and Shannon’s index (I) were 1.335 and 0.367, respectively. The Andaman Sea group showed slightly higher diversity than the Gulf of Thailand group (Andaman Sea: I = 0.382, H_e_ = 0.232, H_o_ = 0.196; Gulf of Thailand: I = 0.353, H_e_ = 0.213, H_o_ = 0.150). Overall, H_o_ (0.173) was lower than H_e_ (0.223), consistent with heterozygote deficiency. PPL was high and comparable between coasts (Andaman Sea 99.66%, Gulf of Thailand 99.89%). For *S. caseolaris*, mean N_e_ and I across coasts were 1.726 and 0.589, respectively. The Andaman Sea group exhibited slightly lower diversity than the Gulf of Thailand group (Andaman Sea: I = 0.554, H_e_ = 0.377, H_o_ = 0.303; Gulf of Thailand: I = 0.624, H_e_ = 0.434, H_o_ = 0.277). Overall, H_o_ (0.290) was lower than H_e_ (0.406), indicating heterozygote deficiency. PPL was high and comparable between coasts (Andaman Sea 98.21%, Gulf of Thailand 100.00%). The inbreeding coefficient (*F_IS_*) was positive on both coasts in both species. Genetic diversity was lower in *S. alba* than in *S. caseolaris* (mean H_e_ = 0.223 vs. 0.406; N_e_ = 1.335 vs. 1.726), with only minor differences between coasts within each species.

## 4. Discussion

Understanding genetic diversity and population structure is essential for developing informed conservation strategies for mangrove genetic resources. In this study, we used SNP markers to evaluate the genetic diversity and population structure of *S. alba* and *S. caseolaris* from the coastal range of Thailand. The observed population stratification offers insight into how geographic configuration and species-specific ecological traits influence evolutionary processes in mangrove systems. STRUCTURE analysis separated *S. alba* populations into three groups (one showing admixture), whereas *S. caseolaris* populations were divided into two major genetic clusters. Importantly, the inferred population structure corresponded closely to the geographical origins, with clear separation between the Andaman Sea and the Gulf of Thailand coasts. Both PCA and the NJ tree supported this pattern, indicating that coastal configuration is a dominant driver of genetic differentiation in both species. Interestingly, one genetic cluster of *S. alba* showed genetic admixture between the two coastal regions, while such an admixture group was not observed in *S. caseolaris*. This contrast may reflect differences in ecological niche and dispersal dynamics between the two species. *S. alba* typically occupies exposed seaward fringe habitats and produces buoyant propagules that may occasionally disperse over longer distances. In addition, anthropogenic translocation associated with mangrove restoration programs, where seedlings are moved across regions to maximize planting success, may have contributed to the observed admixture in *S. alba* populations [57]. In contrast, *S. caseolaris* predominantly occurs in sheltered estuarine environments, where propagule dispersal is likely more localized, resulting in stronger genetic isolation between coastal regions.

High levels of genetic differentiation were observed between subpopulations of *S. alba* (*F*_ST_ = 0.364, *p* < 0.001) and *S. caseolaris* (*F*_ST_ = 0.321, *p* < 0.001), whereas pairwise *F*_ST_ values within the same coastal region were relatively low. This pattern indicates ongoing gene exchange among geographically proximate populations but limited connectivity across the two coasts. The Malay Peninsula likely acts as a major land barrier that restricts oceanic circulation and prevents the dispersal of sea-drifted propagules between the Andaman Sea and the Gulf of Thailand. Similar east–west genetic breaks have been reported in several mangrove species, including *Rhizophora mucronata* [58], *Rhizophora apiculata* [40,58], *Bruguiera parviflora* [38], *Bruguiera cylindrica* [59], *Bruguiera gymnorhiza* [37], and *Ceriops tagal* [39], supporting the Malay Peninsula barrier hypothesis [60]. The relatively low estimates of gene flow (N_m_ = 0.437 in *S. alba* and N_m_ = 0.528 in *S. caseolaris*; Table 1) are consistent with this interpretation.

Isolation-by-distance (IBD) analyses further revealed species-specific patterns of spatial genetic structure. No significant correlation between genetic and geographic distance was detected in *S. alba*, suggesting that spatial separation alone does not explain its genetic structure. In contrast, *S. caseolaris* exhibited significant IBD patterns along both coasts, indicating that gene flow in this species is more sensitive to geographic distance. This pattern is consistent with IBD signals reported in other mangrove species, such as *Rhizophora mangle* [61]. The genetic structure may be influenced by multiple factors, including the Malay Peninsula barrier, geographic distance, ocean currents, sea level changes, climatic variation, and other environmental processes that interact to shape the spatial genetic patterns of mangrove populations in Thailand [27,60,61,62,63,64,65].

While these results provide robust insights into population structure and evolutionary connectivity, it is important to clarify the scope, strengths, and limitations of the genomic approach applied in this study. The RAD-seq approach employed here was designed to efficiently capture genome-wide SNP markers suitable for population-level inference in non-model species, enabling characterization of chromosomal SNP distributions and broad patterns of genomic variation across populations. This approach allowed assessment of SNP recovery across chromosomes and classification of variants according to their genomic positions, thereby providing additional biological context for the observed population genetic structure. However, RAD-seq also has inherent limitations. Because SNP discovery is restricted to regions flanking restriction enzyme cut sites and relies on species-specific reference genomes, the approach is not optimized for comprehensive functional annotation, fine-scale linkage disequilibrium analyses, or direct cross-species comparisons of homologous variants. In addition, this restriction-site–dependent sampling limits the ability to comprehensively capture known adaptive loci previously identified through candidate gene approaches. Consequently, while the present study provides genome-wide insights into population structure and evolutionary connectivity, direct assessment of trait-associated genetic variation will require complementary approaches integrating population genomics with targeted functional analyses. Nevertheless, the RAD-seq dataset generated in this study provides a robust framework for detecting evolutionary subdivision and assessing connectivity among mangrove populations, and represents a valuable resource for future studies that aim to link population genomic patterns with functional and adaptive variation. 

The levels and patterns of genetic variation in *S. alba* and *S. caseolaris* revealed low genetic diversity across species and coastal regions. *S. alba* showed lower genetic diversity than *S. caseolaris* (mean H_e_ = 0.223 vs. 0.406; N_e_ = 1.335 vs. 1.726), consistent with previous studies reporting reduced genetic variability in *S. alba* relative to other mangrove taxa [24]. This may reflect its more restricted ecological niche in exposed seaward zones, which makes the species more vulnerable to demographic bottlenecks and environmental disturbances [66]. In contrast, *S. caseolaris* typically occupies sheltered estuarine habitats, which can support larger and more stable population sizes [67]. Our findings are consistent with reports of low genetic diversity in *S. alba* populations across Sabah, Malaysia (H_e_ = 0.21; Hₒ = 0.21; [68]) and across the Indo–West Pacific (H_e_ = 0.28; Hₒ = 0.26; [27]), although some studies have reported slightly higher values [63]. For *S. caseolaris*, the diversity observed in Thailand was also low and comparable to that reported for populations in Vietnam (I = 0.447; h = 0.300) [28]. Similar patterns of low genetic diversity have also been documented in other mangrove species [69,70,71,72,73,74,75], suggesting that reduced genetic variation may be a common feature of mangrove ecosystems subject to strong environmental filtering and anthropogenic pressure.

Low genetic diversity in *S. alba* and *S. caseolaris* suggests increased vulnerability to multiple biological and environmental stressors. Breeding systems, vegetative reproduction, and propagule dispersal patterns can also influence genetic diversity within mangrove populations [69,76,77]. Additionally, populations of both species are threatened by complex coastal geomorphology and a range of human-induced pressures, including urbanization, wood harvesting, conversion to agriculture and aquaculture, and tourism development [18,78,79]. These factors may accelerate habitat fragmentation and lead to further loss of genetic diversity in *S. alba* and *S. caseolaris* in Thailand. The particularly low diversity observed in *S. alba* demonstrates the need for conservation actions focused on maintaining gene flow, protecting remnant populations, and preventing further fragmentation. From a broader biological and conservation perspective, the pronounced genetic subdivision observed between the Andaman Sea and Gulf of Thailand populations indicates limited evolutionary connectivity between coastal regions. Conservation planning should therefore account for regional genetic differentiation when implementing mangrove restoration programs. Maintaining populations within their respective coastal zones during propagule sourcing may help preserve distinct genetic lineages and reduce the risk of disrupting locally adapted populations. In situ conservation appears appropriate for both species, while carefully guided augmentation using genetically informed sources from high-diversity populations may enhance genetic variation in depleted populations. The use of SNP-based genomic information to guide such management decisions can help prevent unintentional genetic homogenization across regions and support the long-term adaptive potential of mangrove ecosystems under ongoing environmental change.

## 5. Conclusions

This is the first study to investigate the genetic diversity and population structure of *S. alba* and *S. caseolaris* across the coastal range of Thailand using genome-wide SNP markers. Population structure analyses, PCA, and NJ trees showed that *S. alba* populations were clustered into three major groups, whereas *S. caseolaris* populations were clustered into two major groups. These clusters exhibited a clear geographical pattern corresponding to the Andaman Sea and Gulf of Thailand coasts, suggesting that genetic dispersal in both species is strongly influenced by the geographic barrier of the Malay Peninsula. AMOVA and pairwise *F*_ST_ analyses indicated relatively high genetic differentiation between populations, with genetic variation partitioned mainly within populations. Overall, low levels of genetic diversity were observed in both species. Together, these findings provide genome-wide insights into the evolutionary divergence and coastal connectivity of *S. alba* and *S. caseolaris* in Thailand. The clear regional subdivision observed in both species indicates that populations from the two coasts represent distinct evolutionary lineages. Maintaining these lineages in restoration and reforestation efforts will help preserve their adaptive potential, particularly under accelerating coastal change. Therefore, matching planting materials to their native coastal regions may help strengthen the long-term resilience of Thailand’s mangrove forests.

## Figures and Tables

**Table 1:** Analysis of molecular variance (AMOVA) for *S. alba* and *S. caseolaris*. Genetic variance is partitioned among populations and within populations defined by STRUCTURE-inferred populations.

Source of Variation	df	Sum of Squares	Variance Components	Percentage of Variation (%)	Global *F_ST_*
*S. alba*					*F_ST_* = 0.364 **
Among populations	2	16,161.14	128.15	36.43	
Within populations	212	47,191.30	223.65	63.57	
Total	213	63,352.44	351.81		
N_m_ = 0.437					
*S. caseolaris*					*F_ST_* = 0.321 **
Among populations	1	45,544.92	451.572	32.15	
Within populations	260	247,797.39	953.067	67.85	
Total	261	293,342.31	1404.639		
N_m_ = 0.528					

Notes. df: degree of freedom, *F*_ST_: genetic differentiation, Nm: Gene flow ** statistically significance level at 99% (*p* < 0.01). Populations were defined by STRUCTURE-inferred clusters (K = 3 for *S. alba*, K = 2 for *S. caseolaris*).

**Table 2:** Genetic diversity parameters for *S. alba* and *S. caseolaris* by coast and overall, based on 2664 and 5208 SNPs, respectively.

Species	N	N_e_	I	H_o_	H_e_	PPL	*F_IS_*
*S. alba*							
Andaman Sea	43	1.346 ± 0.005	0.382 ± 0.003	0.196 ± 0.002	0.232 ± 0.002	99.66%	0.055 ± 0.006
Gulf of Thailand	64	1.324 ± 0.006	0.353 ± 0.003	0.150 ± 0.002	0.213 ± 0.002	99.89%	0.212 ± 0.005
Overall	107	1.335 ± 0.004	0.367 ± 0.002	0.173 ± 0.001	0.223 ± 0.002	99.77%	0.134 ± 0.004
*S. caseolaris*							
Andaman Sea	34	1.666 ± 0.004	0.554 ± 0.002	0.303 ± 0.002	0.377 ± 0.002	98.21%	0.191 ± 0.004
Gulf of Thailand	97	1.785 ± 0.002	0.624 ± 0.001	0.277 ± 0.002	0.434 ± 0.001	100.00%	0.361 ± 0.004
Overall	131	1.726 ± 0.002	0.589 ± 0.001	0.290 ± 0.002	0.406 ± 0.001	99.11%	0.277 ± 0.003

Notes. N: number of samples, N_e_: number of effective alleles, I: Shannon’s information index, H_o_: observed heterozygosity, H_e_: expected heterozygosity, PPL: percentage of polymorphic loci, *F_IS_*: Inbreeding coefficient.

## Data Availability

Data is contained within the article or Supplementary Materials.

## Supplementary Materials

The following supporting information can be downloaded at https://www.mdpi.com/article/10.3390/biology15020141/s1. Table S1: List of *S. alba* accessions; Table S2: List of *S. caseolaris* accessions; Table S3: List of 2664 SNPs and their characteristics in *S. alba*; Table S4: List of 5208 SNPs and their characteristics in *S. caseolaris*; Table S5: Distribution of SNPs across chromosomes in *S. alba*; Table S6: Distribution of SNPs across chromosomes in *S. caseolaris*; Table S7: Pairwise F_ST_ among genetic clusters of *S. alba* and *S. caseolaris* based on SNP markers; Table S8: Pairwise F_ST_ among sampling locations of *S. alba* and *S. caseolaris*; Figure S1: Distribution of polymorphism information content (PIC) values for (A) 2664 SNP markers among 107 *S. alba* accessions and (B) 5208 SNP markers among 131 *S. caseolaris* accessions; Figure S2: Distribution of SNPs across the chromosomes of the reference genomes of (A) *S. alba* and (B) *S. caseolaris*; Figure S3: Delta K values from STRUCTURE analysis of (A) 107 *S. alba* accessions and (B) 131 *S. caseolaris* accessions.

## Abbreviations

The following abbreviations are used in this manuscript:

SNP	Single-nucleotide polymorphism
RAD-seq	Restriction site-associated DNA sequencing
PCA	Principal component analysis
NJ tree	Neighbour-joining tree
AMOVA	Analysis of molecular variance
IBD	Isolation by distance
PIC	Polymorphism information content
