# Supplementary material for: Genetic Diversity and Population Structure in Two Mangrove Species (Sonneratia alba and Sonneratia caseolaris) Across Coastal Areas of Thailand
Source: Biology (Basel). 2026 Jan 13;15(2):141. doi: 10.3390/biology15020141 (PMC12837700; doi:10.3390/biology15020141)
Supplement: Supplementary file 1 [file biology-15-00141-s001.zip › Supplementary Figure.pdf]

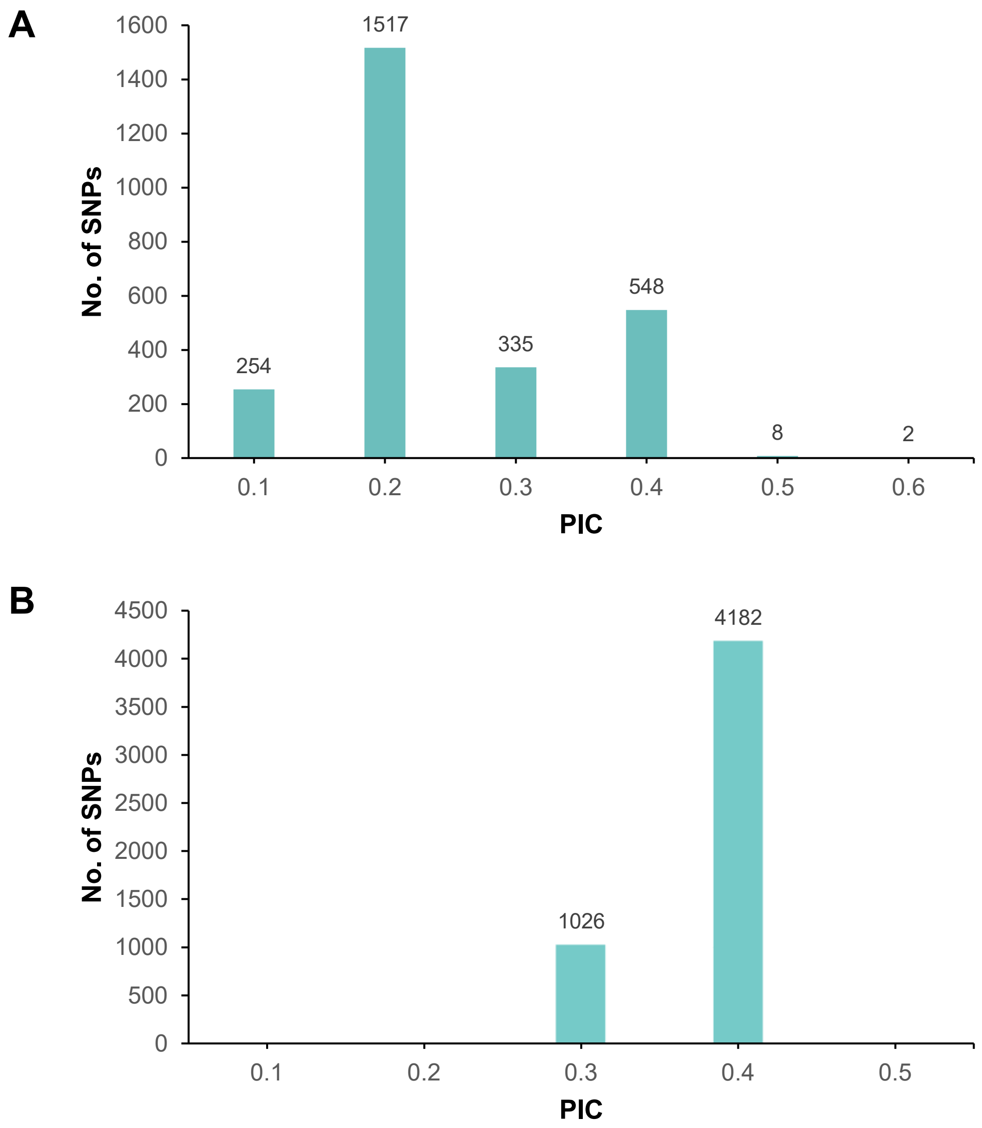

Figure S1. Distribution of polymorphism information content (PIC) values for (A) 2,664 SNP markers among 107 *S. alba* accessions and (B) 5,208 SNP markers among 131 *S. caseolaris* accessions.

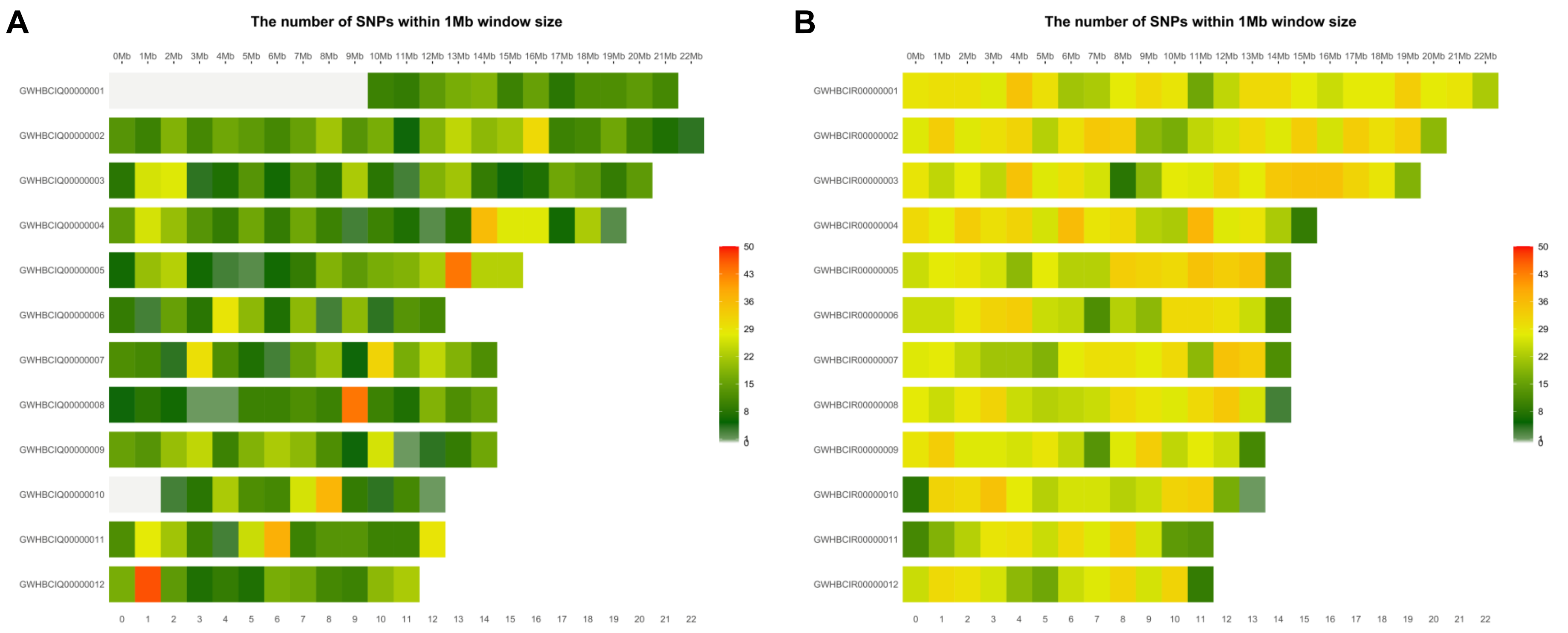

Figure S2. Distribution of SNPs across the chromosomes of the reference genomes of (A) *S. alba* and (B) *S. caseolaris*. Chromosome length is shown along the x-axis and divided into 1 Mb windows. Each horizontal bar represents a chromosome, and colors indicate the number of SNPs per 1 Mb window, ranging from 0 to >50 SNPs.

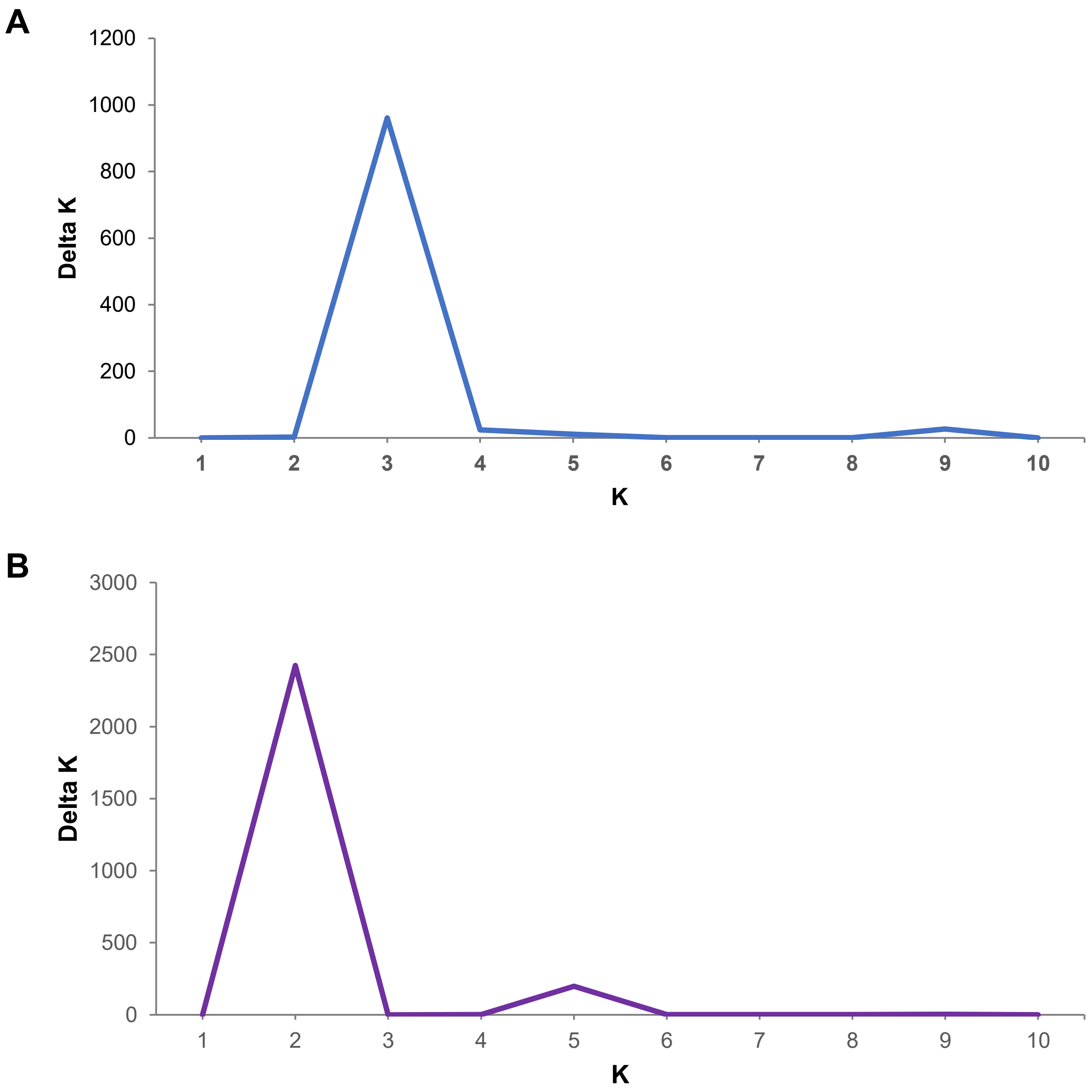

Figure S3. Delta K values from STRUCTURE analysis of (A) 107 *S. alba* accessions and (B) 131 *S. caseolaris* accessions.
